# Postoperative intestinal obstruction caused by staple-related internal hernia after laparoscopic appendectomy: a case report

**DOI:** 10.31744/einstein_journal/2026RC1256

**Published:** 2025-12-11

**Authors:** Francisco Cesar Martins Rodrigues, Rafaela Reynol Rodrigues, Lucas Chiba Kamergorodsky, Gil Kamergorodsky

**Affiliations:** 1 Irmandade da Santa Casa de Misericórdia de São Paulo Department of Gynecology and Obstetrics São Paulo SP Brazil Department of Gynecology and Obstetrics, Irmandade da Santa Casa de Misericórdia de São Paulo, São Paulo, SP, Brazil.; 2 Faculdade de Ciências Médicas da Santa Casa de São Paulo São Paulo SP Brazil Faculdade de Ciências Médicas da Santa Casa de São Paulo, São Paulo, SP, Brazil.; 3 Hospital Israelita Albert Einstein Faculdade Israelita de Ciências da Saúde Albert Einstein São Paulo SP Brazil Faculdade Israelita de Ciências da Saúde Albert Einstein, Hospital Israelita Albert Einstein, São Paulo, SP, Brazil.; 4 Universidade Federal de São Paulo Department of Gynecology and Obstetrics São Paulo SP Brazil Department of Gynecology and Obstetrics, Universidade Federal de São Paulo, São Paulo, SP, Brazil.

**Keywords:** Sutures, Appendectomy, Surgical staplers, Intestinal obstruction, Suture techniques

## Abstract

Postoperative intestinal obstruction is most commonly associated with adhesions but may also arise from unusual causes such as surgical staples adhering to adjacent structures. Although the use of endoscopic staplers in laparoscopic appendectomy is effective and generally safe, it can occasionally result in complications, including intestinal obstruction. We report the case of a 41-year-old woman who underwent surgical treatment for pelvic endometriosis, including appendectomy, and subsequently developed intestinal obstruction caused by an internal hernia formed by the entrapment of a surgical staple from the appendiceal suture line in the jejunal loop. The patient recovered uneventfully after laparoscopic release and removal of staple, with complete resolution of symptoms. This case underscores the importance of vigilance during staple application and reinforces the need for continued improvements in stapling techniques to prevent similar complications.

## INTRODUCTION

The use of endoscopic linear stapling devices in laparoscopic appendectomy surgeries has become increasingly common because of their effectiveness, safety, speed, and ease of manipulation compared with other techniques for closing the appendicular stump.^( 1 )^ Although, in most cases, their use is not associated with morbidity, there have been reports of complications related to the attachment of staples to adjacent viscera, which may lead to intestinal obstruction.

Postsurgical intestinal obstruction is relatively common, occurring in approximately 10% of patients who undergo abdominal surgery.^( 2 )^ However, in appendectomy, it is less frequent, affecting approximately 0.4%–1.54% of patients.^( 3 )^ The role of staples in such events remains poorly understood, with few reports available in the literature. Nonetheless, staples are estimated to cause obstructive complications in approximately 1.8% of laparoscopic surgical procedures.^( 4 )^ Given that postoperative obstruction is most frequently attributed to adhesions,^( 5 , 6 )^ it is important to recognize that less common mechanisms such as staple-related complications, may also contribute and should be considered in the differential diagnosis.

Fourteen case reports have been identified in the literature describing intestinal obstruction caused by surgical staples. Most documented cases result from loose staples remaining within the peritoneal cavity.^( 7 - 17 )^ However, there are a few reports in which functional staples from the suture line have been implicated as the cause of obstruction.^( 18 - 20 )^

We present a case of postsurgical intestinal obstruction caused by an internal hernia formed by the attachment of a staple from the angle of an appendectomy suture line to a jejunal loop.

## CASE REPORT

A 41-year-old female patient diagnosed with deep pelvic endometriosis underwent surgery, including appendectomy, for symptoms that were refractory to clinical management. The appendix was resected because of the presence of an endometriotic lesion, a finding reported in approximately 2.8% of surgical endometriosis cases.^( 21 , 22 )^ Resection was performed using an endoscopic linear stapling device.

**Figure 1:**
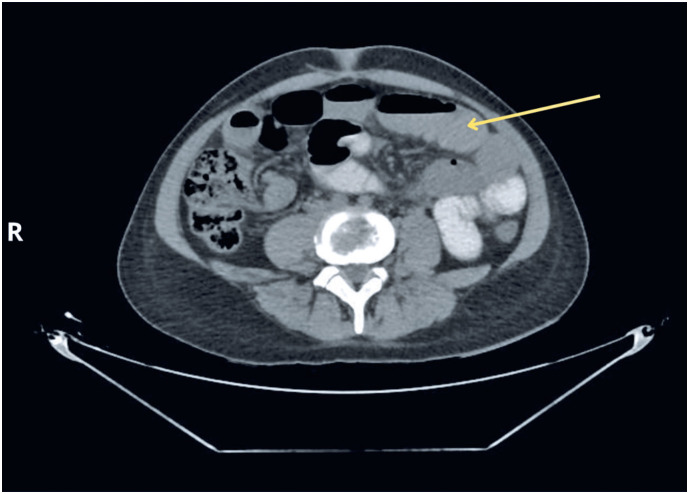
CT-Scan showing air-fluid level and small bowel dilatation

Approximately 12 days after surgery, the patient developed early postoperative intestinal obstruction, presenting with nausea, vomiting, abdominal distension, and colicky pain. Initial management was conservative, consisting of fasting, nasogastric tube insertion, and intravenous hydration, based on the suspicion of postsurgical peritoneal adhesions as the cause of obstruction. However, because the symptoms persisted, computed tomography (CT) was performed, revealing an air-fluid level and small bowel dilatation ( Figure 1 ), findings consistent with intestinal obstruction.

**Figure 2:**
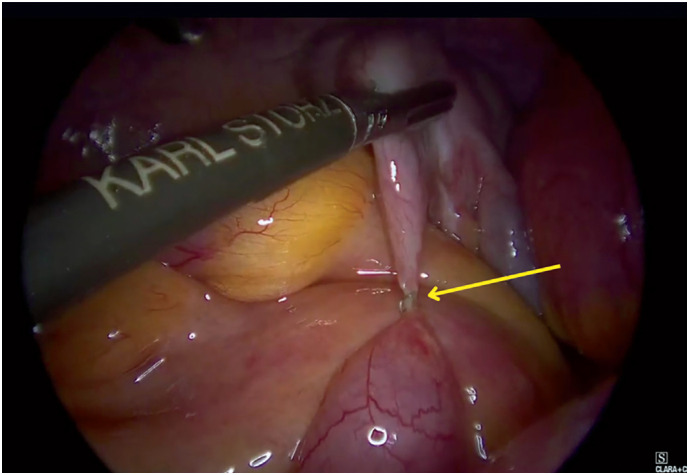
Staple attached to the jejunum

**Figure 3:**
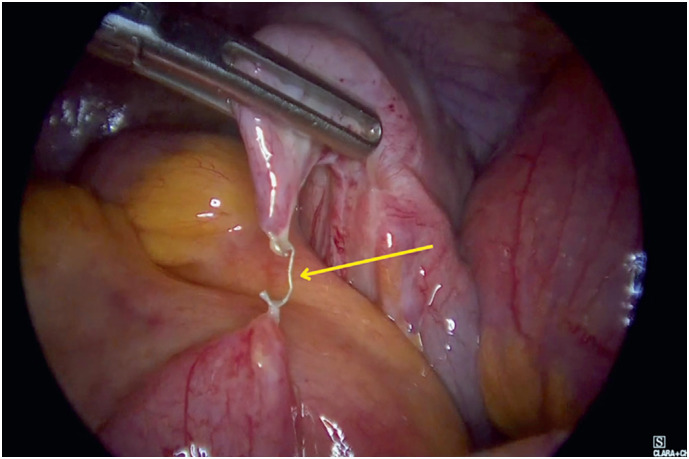
Staple attached to the jejunum after surgical release

Exploratory videolaparoscopy identified an internal hernia caused by the attachment of a surgical staple at the base of the appendiceal suture line, which had encircled the mid-jejunal loop ( Figures 2 and 3 ). The staple was detached from the loop and removed, resolving the obstruction without evidence of intestinal ischemia. The patient recovered uneventfully and was discharged 7 days after the procedure.

The study was approved by the Research Ethics Committee of *Hospital Santa Joana* (CAAE: 79274924.4.0000.5443; #6.837.452).

## DISCUSSION

This case represents an unusual cause of postoperative intestinal obstruction. The final staple at the end of the appendiceal suture line acted as a hook, catching an intestinal loop and predisposing the patient to internal hernia formation and, consequently, mechanical obstruction. In this case, all staples were properly applied and well formed, as confirmed during surgery. Obstruction caused by staples usually occurs early in the postoperative period, as observed in our case, although delayed presentations have also been reported.^( 8 , 13 )^

Although postoperative obstruction may result from various causes, its initial management should always be conservative, whether the obstruction is functional or mechanical. Adhesions are often responsible and typically respond to clinical measures such as fasting, gastric decompression, and analgesia. Given that these are not infectious events, patients can be safely monitored for symptom resolution. When conservative management fails and symptoms persist, surgical intervention becomes necessary, as in the present case. Performing the procedure using a minimally invasive approach allows diagnostic confirmation and facilitates appropriate treatment while minimizing patient morbidity.

Most cases reported in the literature describe events caused by free staples left within the peritoneal cavity,^( 7 - 17 )^ whereas only a few implicate staples from the suture line itself, as in this case.^( 18 - 20 )^ Although three previous reports have documented similar mechanisms, such events remain extremely rare and likely under-recognized. The present case reinforces the importance of including staple-related complications in the differential diagnosis of postoperative intestinal obstruction. It also contributes to the limited literature by illustrating the clinical presentation and successful management of this complication in a unique context—a patient who underwent appendectomy for endometriosis.

Despite the significant benefits and advances of stapling techniques in recent years, this case underscores the need for awareness of potential complications, even when staples are correctly applied. Surgeons should ensure that all loose staples are removed from the cavity, confirm the integrity of the suture line, and verify that the staples are properly formed. Furthermore, manufacturers and device developers should continue to investigate the mechanisms predisposing to such complications and develop preventive solutions.

## DATA AVAILABILITY

The underlying content is contained within the manuscript.
